# Automatic Detection of Mandibular Fractures in Panoramic Radiographs Using Deep Learning

**DOI:** 10.3390/diagnostics11060933

**Published:** 2021-05-22

**Authors:** Dong-Min Son, Yeong-Ah Yoon, Hyuk-Ju Kwon, Chang-Hyeon An, Sung-Hak Lee

**Affiliations:** 1 School of Electronic and Electrical Engineering, Kyungpook National University, 80 Daehakro, Bukgu, Daegu 41566, Korea; forhollow@knu.ac.kr (D.-M.S.); olin1223@ee.knu.ac.kr (H.-J.K.); 2 School of Dentistry, Kyungpook National University, 2177 Dalgubeol-daero, Jung-gu, Daegu 41940, Korea; yayoon@knu.ac.kr (Y.-A.Y.); chan@knu.ac.kr (C.-H.A.)

**Keywords:** mandibular fracture, panoramic radiography, deep learning, object detection, YOLO, YOLO v4, image processing, multi-scale luminance adaptation transform (MLAT), single-scale luminance adaptation transform (SLAT)

## Abstract

Mandibular fracture is one of the most frequent injuries in oral and maxillo-facial surgery. Radiologists diagnose mandibular fractures using panoramic radiography and cone-beam computed tomography (CBCT). Panoramic radiography is a conventional imaging modality, which is less complicated than CBCT. This paper proposes the diagnosis method of mandibular fractures in a panoramic radiograph based on a deep learning system without the intervention of radiologists. The deep learning system used has a one-stage detection called you only look once (YOLO). To improve detection accuracy, panoramic radiographs as input images are augmented using gamma modulation, multi-bounding boxes, single-scale luminance adaptation transform, and multi-scale luminance adaptation transform methods. Our results showed better detection performance than the conventional method using YOLO-based deep learning. Hence, it will be helpful for radiologists to double-check the diagnosis of mandibular fractures.

## 1. Introduction

**Figure 1:**
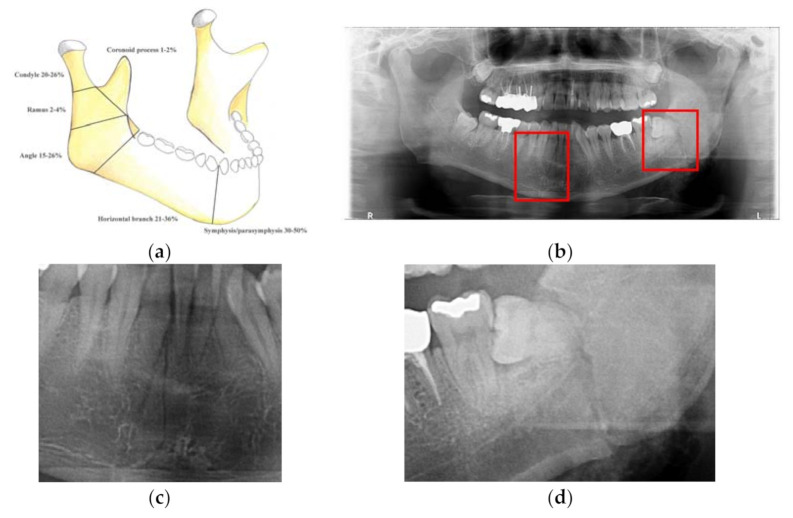
Examples of mandibular fracture: (**a**) anatomic area of mandibular fracture [2], (**b**) mandibular fractures (red boxes) in panoramic radiographs, (**c**) linear fracture on parasymphysis area, and (**d**) shear fracture on angle area.

Mandibular fracture is one of the most frequent injuries in oral and maxillo-facial surgery. Mandibular fractures occur by assault, vehicle accident, fall, among others [1]. They are classified in anatomic areas as follows; symphysis/parasymphysis (30–50%), body/horizontal branch (21–36%), angle (15–26%), ramus (2–4%), condyle (20–26%), and coronoid process (1–2%) as shown in Figure 1a [2]. Mandibular fractures’ shapes are straight, including bending and torsion. These fractures sometimes show shear fractures, as shown in Figure 1b–d [3].

Radiologists mostly diagnose mandibular fractures using cone-beam computed tomography (CBCT) and panoramic radiography. Panoramic radiography is a conventional imaging modality that is the most common way to visualize mandibular fractures [4,5]. Alternatively, CBCT is more capable of observing detailed diseases than panoramic radiography. It has a significantly longer imaging time and higher radiation exposure than panoramic radiography [6]. In panoramic radiography, a routine patient diagnosis is conducted, whereas in CBCT patient diagnosis, it is usually conducted when the patient’s disease is severe. The panoramic radiography has disadvantages of two-dimensional imaging such as patient’s positioning, anatomic noise, superimposition, geometric distortion, and radiographic contrast, as shown in Figure 1c,d. These shortcomings cause inaccurate detection of mandibular fractures. Nevertheless, panoramic radiographs are widely used, thus useful for training and testing datasets of deep learning methods, which are primarily results determined by radiologists.

Nowadays, the deep learning system has been applied in many medical fields. Convolution neural network (CNN) is one of the most popular object detection deep learning methods applied to the medical field. It is configured to be trained by maintaining the association between pixels and the surrounding pixels in the image. We begin the CNN process by generating multiple images that are associated with pixel to pixel from one image. Multiple images are obtained from convolution calculation. By convolution calculation, the adjacent pixel is multiplied by the convolution filter [7]. In practice, deep learning systems have been used in detecting teeth problems using CNN-based methods [8]. These CNN-based methods combine region-based convolution neural network (R-CNN), single-shot multi-box detector, and heuristic methods for detecting teeth, implants, and crowns. In addition, these methods are expected to produce better results by training larger amounts of dental data. That is, the more training data, the better the detection results. Furthermore, there is an automation of tooth segmentation using Mask R-CNN [9]. This automation uses Mask R-CNN without the modification of the deep learning network. The high-quality ground truth and fine-tuning algorithms contribute to higher performance and augmentation techniques, which are rotation, flip, Gaussian blur, and sheared affine transformation. However, it reduces the overfitting of image segmentation. Hence, the training method is an important factor in producing better results.

Recently, another deep learning method, you only look once (YOLO), has also been applied for the detection and classification of breast masses, skin lesion segmentation, and automatic detection of cyst and tumor of the jaw in panoramic radiographs [10,11,12]. The deep learning system for the detection and classification of breast masses is a computer-aided diagnosis (CAD) system based on YOLO. These deep learning methods help in detecting areas of interest in the medical field. They can simultaneously handle the detection and classification of mass for mammography in one framework. However, it is not able to detect the masses of the pectoral muscle and the surrounding dense tissue. To increase the detection accuracy, they use augmentation techniques, such as translation, rotation, and scale changing, to increase the training data.

Generally, these deep learning methods have pre- or post-image processing to increase the size of data. Data augmentation is used to increase the accuracy of detection. Therefore, the selection of an appropriate deep learning method and data augmentation based on the characteristic of symptoms has a great influence on the detection performance. Unlike diseases where specific areas are well-divided in the existing method, it is difficult to learn fracture areas using regional masks. Additionally, when the existing anchor box is used as it is, erroneous detection easily occurs. Therefore, previous techniques are difficult to be applied in detecting the mandibular fracture regions where region segmentation is difficult.

The object detection based on CNN is divided into two; one-stage detection and two-stage detection. One-stage object detection, such as YOLO, is that in which regional proposal and classification are carried out simultaneously. The two-stage object detection, such as Mask R-CNN, executes regional proposal, followed by classification [13]. In general, two-stage object detection shows a better prediction of object detection than one-stage object detection, though it is slower than one-stage object detection. The proposed method uses the recent YOLO v4, one-stage object detection method that allows radiologists to shorten the diagnosis time of mandibular fractures quickly and accurately [14,15,16,17]. As described, our method improves the detection rate of the deep learning system by increasing the amount of data through pre-processing and proliferation of the data in common to learn a vast amount of data. In addition, YOLO is fast and accurate compared to other deep learning methods because it simultaneously conducts detection and classification. Therefore, we used a YOLO deep learning method and pre-processing techniques in the training dataset to detect the mandibular fracture.

Before training the input panoramic radiographs on YOLO v4, the proposed method suggests applying tone mapping operators to input panoramic radiographs. First, adapt image processing to panoramic radiographs, such as gamma modulation and luminance adaptation transform. These pre-processed panoramic radiographs enhance the local contrast, desaturation in bright areas, and the balance between local and global tone rendering for better visibility at the bone border. The enhanced panoramic radiographs are used to increase the accuracy of the detection of mandibular fractures.

In addition, we used two luminance adaptation transforms, single-scale luminance adaptation transform (SLAT) and multi-scale luminance transform (MLAT) [18]. Regional tone mapping operators, such as SLAT and MLAT, convert the original panoramic radiographs with overall dark and irregular background brightness into images with even brightness and better visibility at the bone border to improve detection accuracy. In addition, the training data set contains multi-bounding boxes. The use of multi-bounding boxes is due to the characteristics of mandibular fractures.

The proposed YOLO-based deep learning method is either trained by fractures’ shape or the anatomic areas of mandibular fractures. The comparison of those two training methods demonstrates that the precision score of the fractures’ shape training is lower than the precision score of the anatomic training areas. The characteristic of mandibular fracture has various shapes and sizes. Therefore, the detection of mandibular fractures can be improved by the multi-bounding boxes. We simulated two different categories, two classes related to only the shape of the mandibular fracture, and six classes related to the anatomic area of the mandibular fracture. The purpose of this study is to determine which part of the localization or classification is more influential. The simulation results show that the anatomic area-related six classes pre-processed panoramic radiograph training datasets module presents better predicting mandibular fractures than using only YOLO v4 and other modules which have been simulated.

This study proposed a method that will automatically detect mandibular fractures using deep learning algorithms. Our result will help radiologists not only to diagnose mandibular fractures but to re-confirm their perception of the mandibular fractures. It is expected that automatic detection of mandibular fractures used in YOLO v4 with various techniques will help reduce misdiagnosis.

## 2. Materials and Methods

### 2.1. Related Works

#### 2.1.1. Medical Diagnosis Based on YOLO Deep Learning

**Figure 2:**
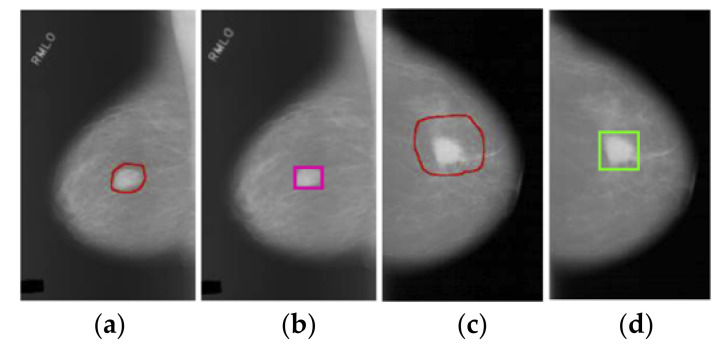
The result of breast mass detection by Mohammed et al. [10]: (**a**) Ground truth of mass (red circle), (**b**) detection by Mohammed et al. (pink box), (**c**) ground truth of a malignant case (red circle), and (**d**) detection by Mohammed et al. (green box). Reprinted with permission from ref. [10]. Copyright 2018, Elsevier.

The YOLO is one-stage object detection and has been utilized in many medical diagnoses. Mohammed et al. presented an automatic detection of the masses in mammogram using YOLO [10]. In the paper, they aimed to establish a novel CAD system based on YOLO. This system contains pre-processing of mammogram, feature extraction, mass detection, and mass classification. The pre-processing part was used for image processing to achieve high performance of the CAD system in the training and testing of datasets. In the mammograms of this training and testing dataset, the shape and position of the malignant mass usually occur in a constant region and shape, as shown in Figure 2. Therefore, we inferred that there is no separate study of localization and shape of objects.

**Figure 3:**
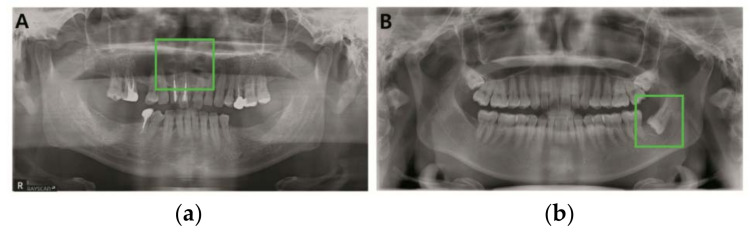
The result of cyst detection (green boxes) by Yang et al. [12]: (**a**) Odontogenic keratocyst and (**b**) dentigerous cyst, both detected by Yang et al.

Another medical diagnosis based on YOLO is by Yang et al. They presented an automated detection of cyst and tumor of the jaw in panoramic radiographs [12]. Note that the odontogenic tumors and cysts are ambiguous radiological features because they do not reveal their characteristic until they reach a certain size. However, they trained many data of the cyst and tumors of the jaw to overcome its flaws. They also tried the YOLO’s localization objects correctness. Odontogenic cysts and tumors appear in various features and borders in panoramic radiographs, as shown in green boxes in Figure 3. Therefore, it is difficult to recognize it in YOLO. Moreover, feature maps of cysts and tumors in YOLO have become too obscure to set the bounding boxes, which may have contributed to the significant false negative (undiagnosed) rate of cysts and tumors.

This research is related to the automatic detection of mandibular fractures in panoramic radiographs. Unlike the masses in the breast, mandibular fractures can occur in random anatomical areas and the shape and size of mandibular fractures are not constant. In addition, mandibular fractures detection has a similar problem in localization and shape as cysts and tumors of the jaw.

#### 2.1.2. The Structure of YOLO

The YOLO is reframed object detection as a single regression problem. That is, YOLO transforms the detection problem into a regression problem. This transformation is fast since a single regression problem does not need a complex pipeline. Additionally, YOLO is faster to process than the R-CNN family because it looks through the entire image, unlike the R-CNN family. Furthermore, YOLO has fewer background errors compared to the R-CNN family. This is because the R-CNN family cannot process the surrounding information of the object. Moreover, one of the characteristics of YOLO is to extract features from the entire image and predict bounding boxes. For each object that presents grid cells on the image, it divides the image into S×S grid, and for each grid cell, it predicts the bounding box’s location and class probabilities. The YOLO feature map tensor is encoded as Equation (1).

**Figure 4:**
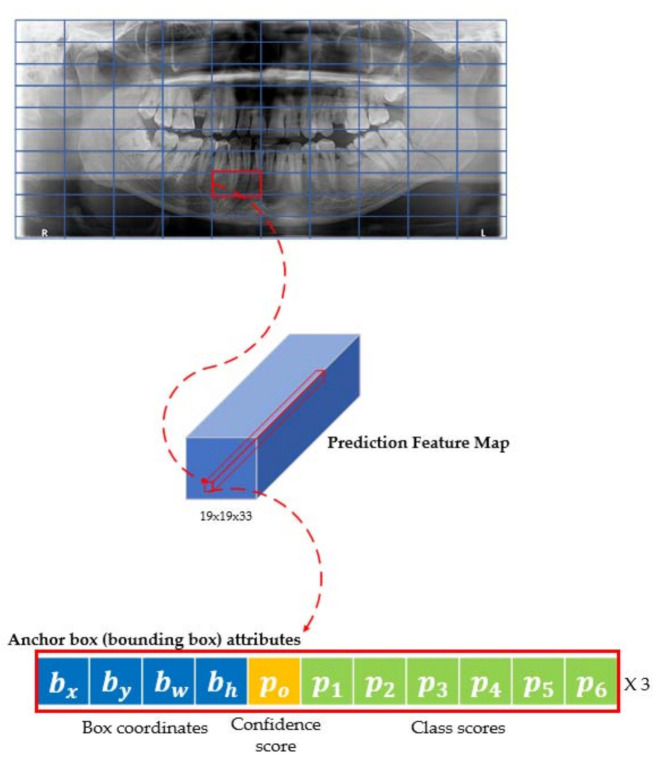
YOLO prediction feature map of the mandibular fracture panoramic radiograph: Red box is a feature map tensor of the mandibular fracture.

(1) feature map tensor=S×S×B×(5+C)

where S is the number of grid cells, B is the number of the bounding boxes (or anchor boxes), and C is the number of class scores. The confidence score depends on whether the object exists inside the bounding box.

(2) $$Confidecne\;score=\mathrm{Pr}\left(Fracture\right)\times IOU_{prediction}^{ground\;truth}$$

where Pr(Fracture) is the probability of the existing mandibular fracture and $IOU_{prediction}^{ground\;truth}$ is the ratio of the intersection over union (IOU). The IOU is the intersection between ground truth and predicted bounding box. The class-specific confidence scores are calculated by confidence score and conditional class probabilities as follows.

(3) $$Class\;specific\;confidence\;score=\mathrm{Pr}\left(Class_{i}|Fracture\right)\times Confidence\;score\;=\mathrm{Pr}\left(Class_{i}\right)\times IOU_{prediction}^{ground\;truth}$$

where $\mathrm{Pr}\left(Class_{i}|Fracture\right)$ is the conditional class probability of ith Class. The class-specific confidence score is multiplied by the confidence score and conditional class probability. This process is shown in Figure 4.

YOLO has been developed from one to four versions. YOLO v1 used bounding boxes to solve a regression problem directly, whereas YOLO v2–v4 used anchor boxes, instead of bounding boxes to easily solve a regression problem. YOLO v2 adapted batch normalization instead of drop-out, anchor boxes, direct location prediction, and multi-scale training methods to improve detection. YOLO v3 adapted residual block to build deep network layers and predictions across scales. The predictions across scales are similar to feature pyramid networks [19]. It expects predicting boxes in three scales of the feature map. The feature map from the beginning of predictions across scales concatenates the up-sampling feature map. This process of concatenation can extract meaningful information from the previous layer and fine-grained information from the current layer. Then, add a convolution layer to handle the concatenated feature map. The same procedure is executed to predict the final scale of the feature map. Based on this process, the prediction of the third scale utilizes the current information from all previous layers and information from the third scale. The anchor boxes in the three scales of the feature map are calculated by k-means clustering. Notably, there are three anchor boxes in each of the three feature maps.

**Figure 5:**
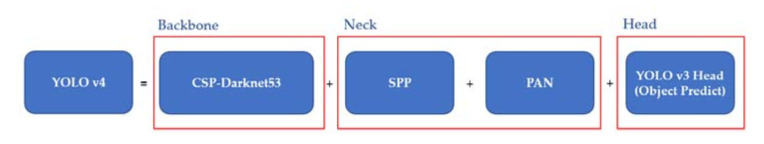
The brief structure of YOLO v4.

**Figure 6:**
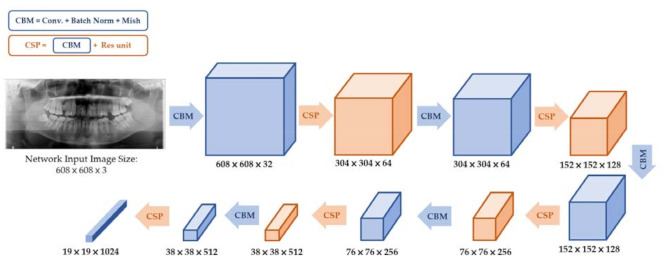
YOLO v4 backbone structure: Cross-stage-partial-connection-Darknet53.

**Figure 7:**
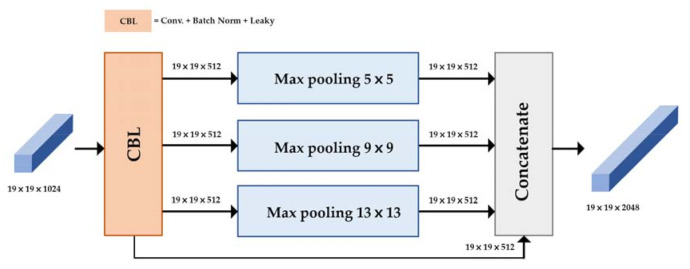
YOLO v4 neck structure: Spatial pyramid pooling layer.

**Figure 8:**
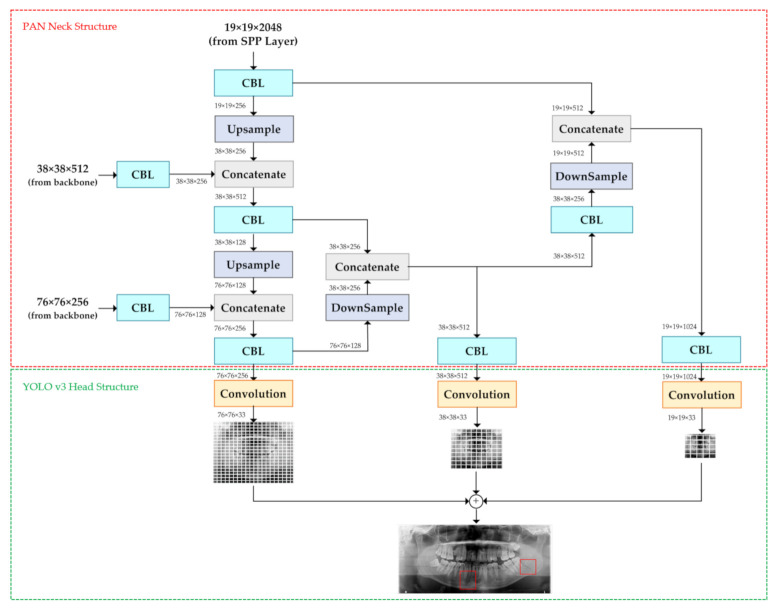
YOLO v4 path aggregation network neck and head structure.

The YOLO v4 adapted new techniques such as cross-stage-partial-connection (CSP) [20], spatial pyramid pooling (SPP) [21], and path aggregation network (PAN) [22] to improve YOLO v3. The YOLO v4 was divided into three parts: backbone, neck, and head, as shown in Figure 5. The backbone structure consists of CSP-Darknet53, which is shown in Figure 6. The neck structure consists of SPP and PAN. Head structure consists of YOLO v3 prediction, which is the same as YOLO v3 architecture. Neck and head structure in YOLO v4 are shown in Figure 7 and Figure 8.

Furthermore, YOLO v4 used new techniques of data augmentation such as CutMix [23] and Mosaic [17] for training the datasets to improve detection ability. The deeper the CNN layers, the higher the object detection capability. Thus, YOLO v4 applied CSP-Darknet53, which is an improvement over Darknet53 shown in Figure 6. The CSP-Darknet53 changed the Mish activation function instead of the leaky-Relu activation function. Additionally, it adapted CSP to make deeper CNN. Again, CSP consists of convolution, residual unit, and concatenation, whereas CSP-Darknet53 does not need to use the bottleneck layers because only half of the feature maps pass through the residual block. It means that in CSP-Darknet53, the residual structure is mapped directly from the previous feature map layer to the latter feature map layer without convolution, and it is helpful for training and feature extraction.

The neck structure includes two different methods, SPP and PAN. Four layers are concatenated in SPP. These four layers are generated by 1×1, 5×5, 9×9, and 13×13 max pooling layers. The max-pooling layer extracts the most significant contextual features, and it increases the receptive field of the backbone feature effectively. Therefore, it can be a powerful method for feature extraction. In Figure 7, we show the SPP in the mandibular fracture YOLO v4 module. In Figure 8, we show how PAN can repeatedly extract features by up-sampling and stacking and then down-sampling and stacking. The aim of PAN is to improve information flow in a proposal-based instant segmentation framework. It enhances the entire feature layer with accurate localization signals of the lower layers by bottom-up path augmentation, which is used for information flow between lower layers and topmost features [22].

Head structure in mandibular fracture YOLO v4 module followed the YOLO v3. After passing through backbone and neck structure, YOLO v4 produced three feature maps, 19×19×33, 38×38×33, and 76×76×33, whenever the module had six classes, as shown in Figure 8. The predicted bounding box is the same as predictions across scales in YOLO v3. The predicted bounding box is marked as red boxes which are shown in Figure 8. The prediction of the third scale (76×76×33) utilized the current information from the previous layers (19×19×33, 38×38×33) and information from the third scale. The predicted bounding box prior to performing non-maximum suppression (NMS) had multiple predicted bounding boxes for one class. Therefore, NMS was used to reduce these multiple predicted bounding boxes. In the mandibular fracture YOLO v4 module, greedy NMS was used because it obtained the best performance when using average precision as an evaluation score [24]. This head structure provided more accurate mandibular fracture detection, especially in small fractures.

### 2.2. Proposed Methods

#### 2.2.1. Data Augmentation

Data augmentation is an important part of the deep learning system. The more training datasets, the more possibility of accurate detection. Therefore, training datasets is an important procedure. Typical data augmentation methods are applied to rescale, flip, and switch targets to increase the number of datasets. However, pre-processing for enhanced data is also one of the methods for data augmentation. For example, the radiographs are dark, and some areas are saturated. If suitable image processing is applied to the radiographs, fractures may be revealed and the detection performance for these will be improved. Thus, before training data, local tone improvement processing is required for the radiographs of various brightness.

This part describes the pre-processing methods to be applied to augment training datasets, which are gamma modulation, luminance adaptation transform, and extended multi-anchor boxes. First, gamma modulation is presented for various brightness background data without over- or under-saturations. It can generate luminance augmentation to train a wide range of brightness information. In the first step, we used three global gamma values: gamma value=γ,(γ=[1.0, 1/0.6, 1/0.3]).

(4) $$O={(\frac{I}{255})}^{\gamma }\times 255$$

where O is an output image of gamma modulation and I is an input image. These gamma values darken the images to accurately represent fractures. If the gamma value is less than 1, the images are brighter and more saturated, making it difficult for the image to recognize fractures.

**Figure 9:**
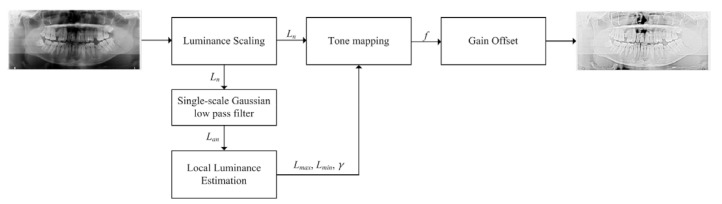
Single-scale luminance adaptation transform.

However, the gamma modulation module tends to detect normal regions that do not have fractures. Thus, we additionally applied luminance adaptation transforms to the gamma shifted training dataset. There are two types of the luminance adaptation transform, SLAT and MLAT [18]. SLAT has two main processes, local tone mapping in the luminance channel and chrominance compensation in the chrominance channel. Since the panoramic radiographs are only grayscale images, only the local tone mapping of the luminance channel is considered in the SLAT process. As the second step for luminance augmentation, the luminance adaptation transform (LAT) process is shown in Figure 9. The SLAT adjusts visually compensated gamma values according to local adaptation luminance level. The luminance level is divided into minimum luminance level and maximum luminance level.

The luminance scaling normalizes the luminance channel up to 100 ($L_{n})$ because local luminance estimation is designed on the condition of the adaptation luminance under surround luminance, 100$\;\mathrm{cd}/m^{2}$. The single-scale Gaussian low pass filter makes a surround image ($L_{an})$, which supposes the adaptation luminance condition.

(5) $$L_{min}=0.0212+0.0185L_{an}^{1.0314}$$

(6) $$L_{max}=25.83+30.82L_{an}^{0.6753}$$

where $L_{min}$ is minimum luminance level, $L_{max}$ is maximum luminance level, and $L_{an}$ is normalized adaptation luminance, which is calculated from the Gaussian low pass filter.

The local visual gamma value, which can affect SLAT, is based on Bartelson–Breneman’s brightness function [25].

The SLAT enhances the local contrast and desaturation in bright area.

(7) $$\gamma =0.444+0.045\ln\left(L_{an}+0.6034\right)$$

where *γ* is the local visual gamma value and $L_{an}$ is normalized adaptation luminance. This gamma value is fixed on Bartelson–Breneman’s brightness function curve.

(8) $$f={\left|\frac{L_{n}-L_{min}}{L_{max}-L_{min}}\right|}^{\gamma }$$

where $L_{n}$ is the normalized luminance input image, $L_{min}$ is the minimum luminance level, $L_{max}$ is the maximum luminance level, and *γ* is the visual gamma.

(9) $$I_{gain}=\frac{R_{cs}}{f_{max}-f_{min}},...\;I_{offset}=\frac{R_{cs}f_{min}}{f_{max}-f_{min}}$$

where $R_{cs}$ is the intensity range of the selected color space, $f_{max}$ is the maximum value of (8), and $f_{min}$ is the minimum value of (8).

(10) $$SLAT=I_{gain}f+I_{offset}$$

Based on the result obtained from these calculations, *SLAT* enhances the local contrast and desaturation in bright area. 

The MLAT is the sum of several SLATs with different surrounding images. Multi-scale related methods require multiple surroundings for the balance between local and global tone rendering in SLAT. It should be noted that MLAT consists of a weighted sum of SLATs using three different scales of low pass filters. The scales of filters are 15, 80, and 250.

(11) $$MLAT=\sum_{1}^{N}w_{n}SLAT_{n}$$

where $SLAT_{n}$ is nth single luminance adaptation transform and $w_{n}$ is nth weighting factor of scales. By this, we observed that *MLAT* improved local and global rendering, and increased both detail qualities and tonal rendition enhancement.

**Figure 10:**
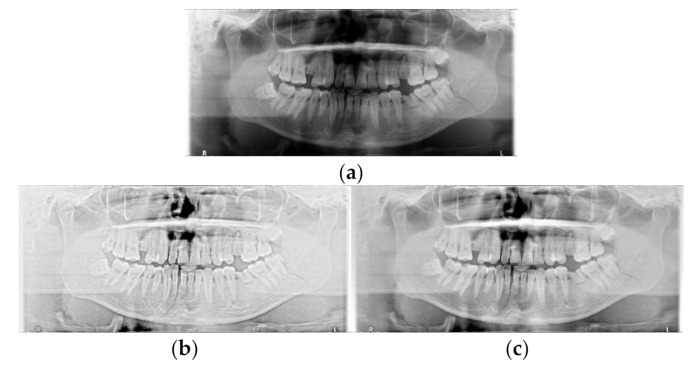
The comparison between original panoramic radiograph, single-scale luminance adaptation transform (SLAT), and multi-scale luminance adaptation transform (MLAT) panoramic radiograph: (**a**) Original, (**b**) SLAT, and (**c**) MLAT.

In conclusion, SLAT and MLAT are applied to clearly show mandibular fractures in panoramic radiographs as shown in Figure 10. SLAT is used for local boundary enhancement by applying a single Gaussian filter. In MLAT, an overall tone compression technique using multiple Gaussian filters is applied to mitigate the local noise amplification of SLAT. Moreover, the SLAT images are useful for searching detailed feature parts while MLAT helps in providing useful images for searching severe fracture areas. Therefore, the purpose of SLAT and MLAT image processing is to increase the detection capability for the YOLO deep learning method.

The last data augmentation method is to apply bounding boxes of different sizes to all training datasets. Since the training datasets are a small amount of radiographs, the data should be effectively increased. The multiple bounding boxes are suitable for data augmentation. We used a multi-bounding box because the characteristics of the mandibular fracture have various shapes and sizes. The size of the multiple bounding boxes is set at 0.7 times and 1.6 times of the reference bounding box, and there are three bounding boxes per one fracture.

These modules are pre-trained with only 54 panoramic radiographs for the trained dataset to compared only pre-process performance. These four modules, which use only 54 panoramic radiographs for the training dataset, perform worse than those that use 360 panoramic radiographs as the training dataset module of the proposed method. However, it is easy to compare pre-process performances. The precision and recall scores of each module for 45 panoramic radiographs test datasets are shown in Table 1.

**Figure 11:**
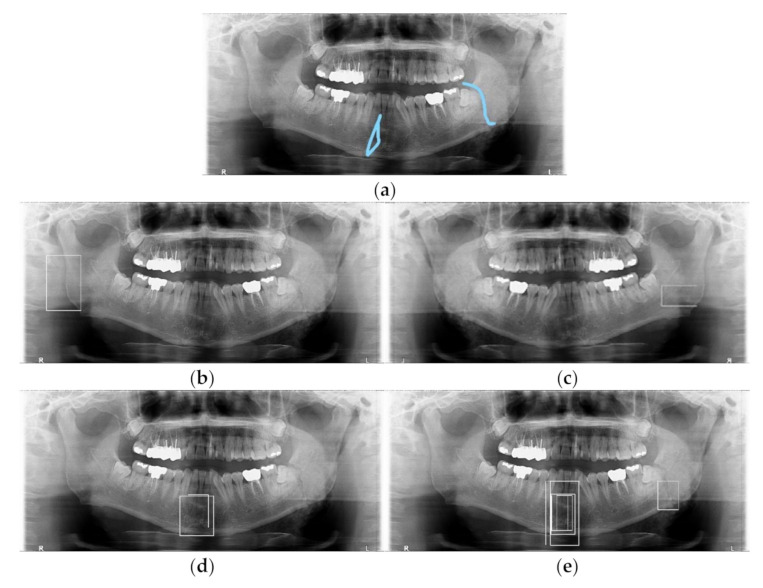
The comparison between different four modules for mandibular fracture detection (white boxes): (**a**) Diagnosed radiographs (blue lines) by a radiologist, (**b**) Original module, (**c**) Gamma modulation module, (**d**) Luminance adaptation transform module, and (**e**) The proposed module.

The original module is not able to detect fractures in most cases. The gamma modulation module is better than the original module and has less precision score than the original module. The luminance adaptation transform can detect mandibular fractures relatively well but the precision and recall scores do not exceed 0.5. The proposed pre-processing module can detect mandibular fractures better than other modules. Thus, we decided to use LAT with gamma modulation and a multiple box module. The comparison of those four modules’ mandibular fracture detection results is shown in Figure 11.

#### 2.2.2. Training and Detection Process

Before training the dataset, the data to be trained should be processed using luminance adaptation transforms, gamma modulation, and multiple bounding boxes. After pre-processing, the datasets are then trained by YOLO v4. The training parameters of YOLO v4 are shown in Table 2.

The YOLO v4 has its own data augmentation options, such as angle, saturation, exposure, hue, and mosaic, which combines four training images to one in certain ratios. Since the proposed method is used in six classes for detecting mandibular fractures, max batches (or iteration) are set to 12,000 and this iteration can be calculated with approximately 711 epochs.

During 12,000 iterations, we were able to obtain the best weight file for mandibular fracture detection. To compare the performance of the six-class SLAT and MLAT module, we also trained two classes of SLAT and MLAT modules under the same conditions. The six classes’ modules are related to the anatomic area of the mandibular fracture (parasymphysis, body, angle, ramus, condyle, and coronoid) whereas two classes of modules were related to the form of the mandibular fracture (shear fracture and linear fracture).

**Figure 12:**
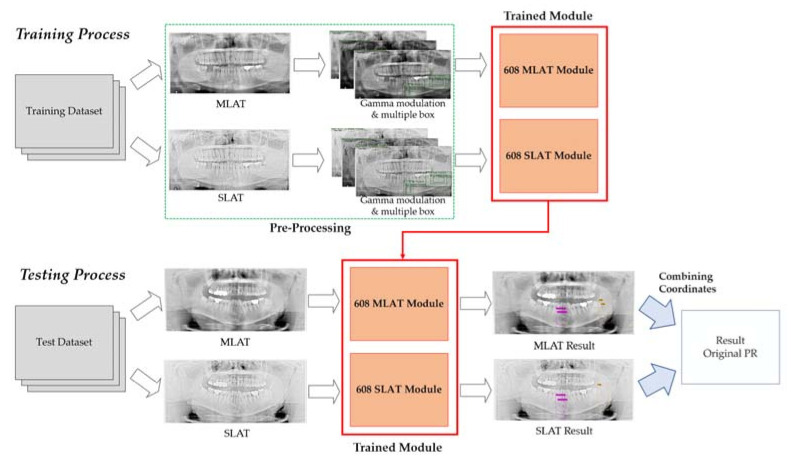
The proposed method’s block diagram of the diagnosis process in YOLO v4.

In the training process, the dataset to be trained was subjected to LAT processing. After the LAT process, the images went through the process of gamma modulation and multiple boxes to reveal the mandibular fracture. After the pre-processing progress, this dataset was trained by the YOLO v4 deep learning network. All images were set to 608×608 resolution and went through a convolution network for the feature map to be extracted. In the testing process, we applied luminance adaptation transform to the test dataset as well. After testing the dataset using both MLAT and SLAT modules, which were trained by the YOLO v4 deep learning network, we obtained coordinates to detect mandibular fractures. When combined with MLAT and SLAT predicted boxes, which are pink and yellow boxes, both coordinates could be applied to the original panoramic radiographs to show radiologists where the fracture was located. The entire process of the proposed method is shown in Figure 12.

#### 2.2.3. Performance Evaluation Metrics

The proposed method was presented as an indicator for object detection evaluation and classification performance on three evaluation metrics, precision, recall, and F1 score. We measured 60 test images of panoramic radiographs by these three metrics.

(12) $$Precision=\frac{TP}{TP+FP}=\frac{Detection}{Detection+Misdetection}$$

(13) $$Recall=\frac{TP}{TP+FN}=\frac{Detection}{Detection+Undetection}$$

(14) $$F1\;score=\frac{2\times \left(Recall\times Precision\right)}{Recall+Precision}$$

where TP, FP, and FN symbolize the true positive, false positive, and false negative. Detection is accurate detection of mandibular fractures, Misdetection is the detection of objects other than mandibular fractures, and Undetection is the detection of nothing. If the recall score was higher, the precision score was low, so it was not possible to determine whether the precision score or the recall score was better. Therefore, it was possible to determine the better object detection with the *F*1 score metric. The *F*1 score is the harmonic mean of precision and recall scores, which we can use to compare better object detection results. Furthermore, the accuracy score, which was frequently used in evaluation metric for object detection, was not available for the proposed method performance evaluation metric. The reason for not using the accuracy score, related to TN (true negative), is that we do not know where fractures will occur in anatomic regions of the mandible.

## 3. Results

### 3.1. Deep Learning System and Dataset

The deep learning system was implemented on a PC with an Intel i7-9700K processor, 32GB RAM, NVIDIA TITAN RTX and a window version of YOLO v4. For the simulation, panoramic radiographs of 420 mandibular fracture patients were used, which consisted of 360 panoramic radiographs of training datasets and 60 panoramic radiographs of test datasets. The resolution of panoramic radiographs was 2228×1244 to 2972×1536 pixels.

### 3.2. Detection Results

We evaluated and compared the detection performance of the proposed method and the deep learning results of various methods. For the classification of training datasets in the proposed model, learning was conducted based on classes of two different sizes related to the shape of fractures or anatomic mandibular fracture regions. We set shear fracture and linear fracture for two classes of shape difference and parasymphysis, body, angle, ramus, condyle, and coronoid for six classes of the anatomic region. In this comparison, we used 360 panoramic radiographs with the proposed pre-processing, such as SLAT, MLAT, gamma modulation, and multiple boxes. We obtained a total of 1080 images for training datasets. We tested 60 panoramic radiographs of test images and a total of 97 mandibular fractures in test datasets.

**Figure 13:**
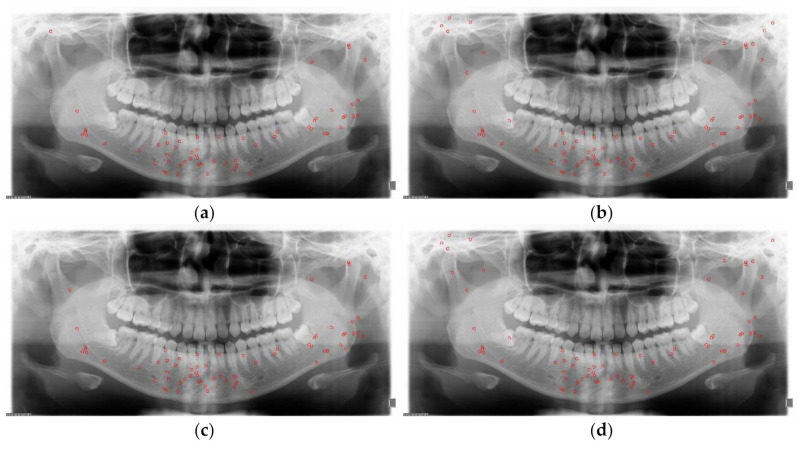
The two-class and six-class comparison of correct diagnosed distribution (red circles) of mandibular fractures: (**a**) two-class single-scale luminance adaptation transform (SLAT), (**b**) six-class SLAT, (**c**) two-class multi-scale luminance adaptation transform (MLAT), and (**d**) six-class MLAT.

**Figure 14:**
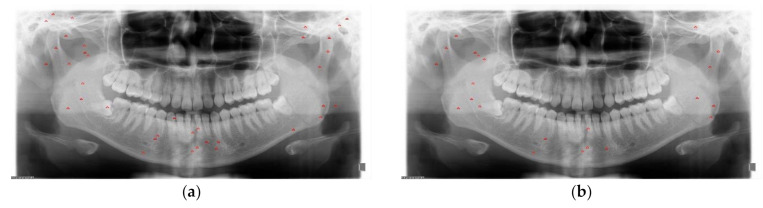

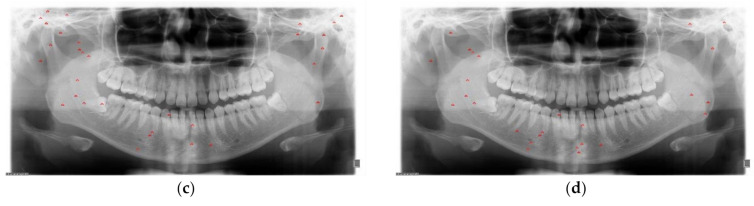
The two-class and six-class comparison of undiagnosed distribution (red triangles) of mandibular fractures: (**a**) two-class single-scale luminance adaptation transform (SLAT), (**b**)six-class SLAT, (**c**) two-class multi-scale luminance adaptation transform (MLAT), and (**d**) six-class MLAT.

The diagnosed and undiagnosed distribution plots of mandibular fractures are shown in Figure 13 and Figure 14. The distribution plots show that six classes of SLAT and MLAT modules have better detection precision. The six-class SLAT and MLAT modules have a less undiagnosed distribution of mandibular fracture than the two-class SLAT and MLAT modules. In these plot images, the six classes as anatomic region classification modules can reduce the chronic problem of localization errors in YOLO v4.

**Figure 15:**
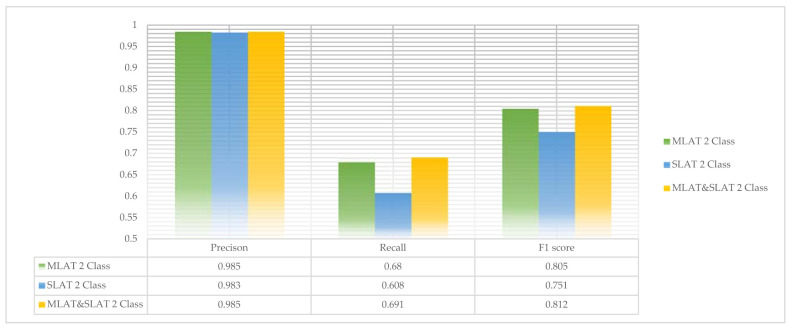
The comparison of multi-scale luminance adaptation transform (MLAT), single-scale luminance adaptation transform (SLAT), and MLAT and SLAT in two-class.

The scores of two-class modules are shown in Figure 15. In two-class modules, our recall scores are less than 0.7. That is, the probability of detecting a fracture is less than two-thirds. However, the precision score is almost 1. What this means is that if we use this module to detect a mandibular fracture, there is almost no error. The MLAT and SLAT module is the highest recall score and F1 score. This module is the complementary module of the MLAT and SLAT modules, which is the best of the three modules.

**Figure 16:**
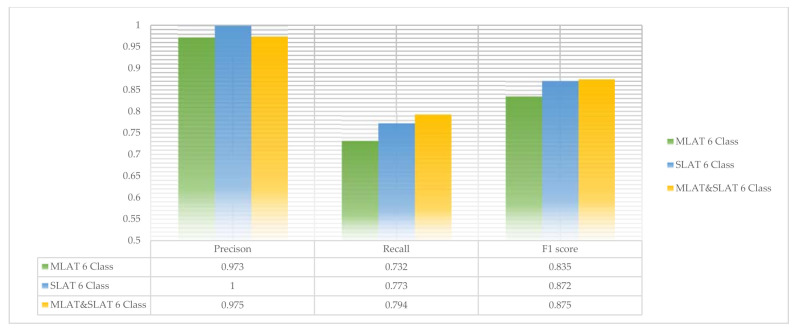
The comparison of multi-scale luminance adaptation transform (MLAT), single-scale luminance adaptation transform (SLAT), and MLAT and SLAT in six-class.

**Figure 17:**
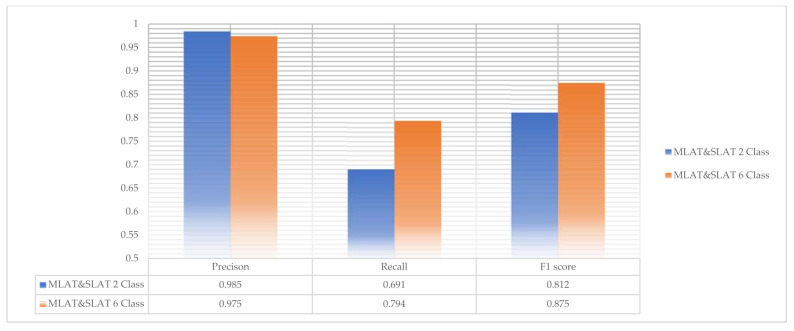
The comparison of two-class and six-class multi-scale luminance adaptation transform and single-scale luminance adaptation transform modules.

The scores of six-class modules are shown in Figure 16. In six-class modules, the recall and F1 scores are higher than two-class modules. That is, six-class modules can detect more mandibular fractures than two-class modules. Even though six-class SLAT modules have no error for detecting mandibular fractures, the total precision score in the six-class MLAT and SLAT module is lower than the two-class modules. But this difference does not have much effect. In conclusion, six-class MLAT and SLAT modules have better performance to detect mandibular fractures. The comparison of two-class and six-class MLAT and SLAT modules’ scores is shown in Figure 17.

## 4. Discussion

The experiments have provided training directions for two different classes, the shape of mandibular fractures and the anatomical region of mandibular fractures. The results presented that the classification based on the anatomical region of mandibular fractures showed better performance than the classification based on the shape of mandibular fractures. The evaluation using metric scores, such as precision, recall, and F1 scores, helped us compare detection performances.

**Figure 18:**
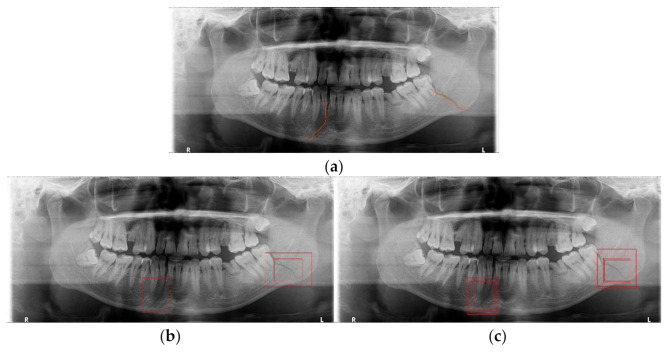
The mandibular fractures diagnosis comparison: (**a**) diagnoses mandibular fractures (orange lines) by radiologist, (**b**) mandibular fracture detection (red boxes) of two-class multi-scale luminance adaptation transform (MLAT) and single-scale luminance adaptation transform (SLAT) module, and (**c**) mandibular fracture detection (red boxes) of six-class MLAT and SLAT module.

**Figure 19:**
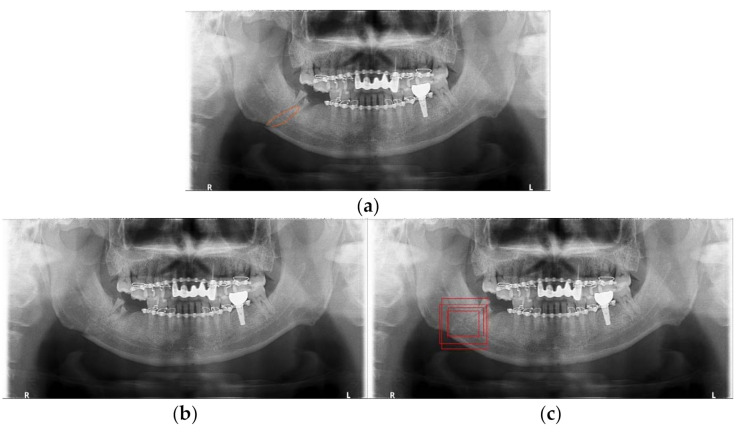
The mandibular fractures diagnosis comparison: (**a**) diagnoses mandibular fractures (orange lines) by radiologist, (**b**) mandibular fracture detection (red boxes) of two-class multi-scale luminance adaptation transform (MLAT) and single-scale luminance adaptation transform (SLAT) module, and (**c**) mandibular fracture detection (red boxes) of six-class MLAT and SLAT module.

**Figure 20:**
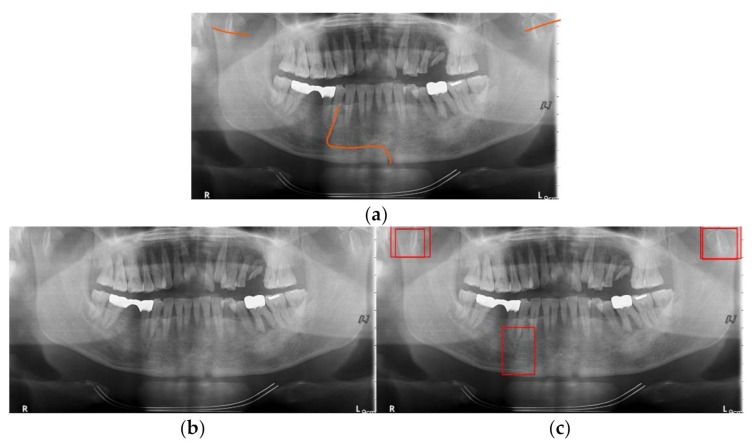
The mandibular fractures diagnosis comparison: (**a**) diagnoses mandibular fractures (orange lines) by radiologist, (**b**) mandibular fracture detection (red boxes) of two-class multi-scale luminance adaptation transform (MLAT) and single-scale luminance adaptation transform (SLAT) module, and (**c**) mandibular fracture detection (red boxes) of six-class MLAT and SLAT module.

In Figure 18, Figure 19 and Figure 20, we show some results of MLAT and SLAT modules and compare them with six-class and two-class modules. The reference panoramic radiographs, which are diagnosed by a specialist in oral maxillofacial radiology, shows the mandibular fractures’ correct position. As for the relatively distinct fracture shape in Figure 18, both two-class and six-class MLAT and SLAT modules can detect correct mandibular fractures’ location, but multi-detection boxes show more weighted results. However, in areas where it is difficult to distinguish the surrounding bone tissue from the anglesite of Figure 19, two-class modules cannot detect a mandibular fracture in the angle position of the mandible. Additionally, in Figure 20, the case of including fractures in the areas of the ramus and condyle sites where it is difficult to be distinguished, six-class modules can detect all positions of mandibular fractures. Moreover, the six-class module tends to detect the upper part of the mandible (condyle area of mandible) well. Therefore, in many cases where visual identification is difficult, the six-class MLAT and SLAT module could be a better choice than the two-class MLAT and SLAT module. The proposed method can be used for detecting mandibular fractures, also for a bone healing process after a surgical operation. Since radiologists can diagnosis a bone healing state after the post-operation in panoramic radiographs [26], so the proposed method can be used to evaluate osteotomies performance in panoramic radiography.

## 5. Conclusions

This paper presents an automatic detection method of mandibular fractures based on the YOLO v4 deep learning model. In general, the original panoramic radiographs are dark and mandibular fractures in panoramic radiographs have severe curvature characteristics at the background level. Therefore, if the existing YOLO-based detection learning is used as it is, detailed fracture identification is impossible.

Therefore, we suggested the data augmentation and pre-processing techniques for the training dataset and test dataset. Gamma modulation, SLAT, and MLAT pre-processing methods showed enhanced detection performance for mandibular fractures with unspecified shapes and areas. To increase the accuracy score of mandibular fracture detection, it is necessary to increase the training data sizes. Thus, we applied multiple boxes on the training dataset to complement that of the small dataset. Additionally, multiple boxes are helpful to detect various sizes and shape of mandibular fractures.

The conclusion derived from the comparison of simulations is that using the six-class module with the combined MLAT and SLAT module results in an effective performance for mandibular fracture detection. The limitation of the proposed method is that we used only panoramic radiography. Panoramic radiography, which is a two-dimensional imaging of mandibular fractures, is usually limited to isolated lesions. However, CT has no overlap between the different anatomic structures [2]. Thus, in the case of multiple facial fractures or comminuted fractures, CT should be diagnosed rather than panoramic radiography to identify the fractures more accurately. Due to these fractures, future works will require the study of mandibular fracture detection with a mixture of panoramic radiography and CT.

It is our hope that the proposed deep learning model will help radiologists and dentists diagnose mandibular fractures.

## Figures and Tables

**Table 1:** The comparison of pre-process performance using precision and recall scores.

	Original	Gamma Modulation	Luminance Adaptation Transform	Proposed Pre-Processing
Precision	0.375	0.341	0.441	0.570
recall	0.048	0.222	0.474	0.714

**Table 2:** The parameters of the proposed method in YOLO v4.

Option	Set Value
Batch size	64
Subdivision	16
Resolution	608 × 608
Momentum	0.949
Decay	0.0005
Learning rate	0.0001
Angle	180
Saturation	1
Exposure	1.5
Hue	0
